# Within-Host Spatiotemporal Dynamics of Plant Virus Infection at the Cellular Level

**DOI:** 10.1371/journal.pgen.1004186

**Published:** 2014-02-27

**Authors:** Nicolas Tromas, Mark P. Zwart, Guillaume Lafforgue, Santiago F. Elena

**Affiliations:** 1 Instituto de Biología Molecular y Celular de Plantas, Consejo Superior de Investigaciones Científicas-UPV, València, Spain; 2 The Santa Fe Institute, Santa Fe, New Mexico, United States of America; Université Paris Descartes, INSERM U1001, France

## Abstract

A multicellular organism is not a monolayer of cells in a flask; it is a complex, spatially structured environment, offering both challenges and opportunities for viruses to thrive. Whereas virus infection dynamics at the host and within-cell levels have been documented, the intermediate between-cell level remains poorly understood. Here, we used flow cytometry to measure the infection status of thousands of individual cells in virus-infected plants. This approach allowed us to determine accurately the number of cells infected by two virus variants in the same host, over space and time as the virus colonizes the host. We found a low overall frequency of cellular infection (<0.3), and few cells were coinfected by both virus variants (<0.1). We then estimated the cellular contagion rate (*R*), the number of secondary infections per infected cell per day. *R* ranged from 2.43 to values not significantly different from zero, and generally decreased over time. Estimates of the cellular multiplicity of infection (*MOI*), the number of virions infecting a cell, were low (<1.5). Variance of virus-genotype frequencies increased strongly from leaf to cell levels, in agreement with a low *MOI*. Finally, there were leaf-dependent differences in the ease with which a leaf could be colonized, and the number of virions effectively colonizing a leaf. The modeling of infection patterns suggests that the aggregation of virus-infected cells plays a key role in limiting spread; matching the observation that cell-to-cell movement of plant viruses can result in patches of infection. Our results show that virus expansion at the between-cell level is restricted, probably due to the host environment and virus infection itself.

## Author Summary

A great deal is understood about how a virus infects an individual cell and manages to replicate. Patterns of disease progression in plant and animal hosts, such as virus titers and the appearance of symptoms, have also been described in great detail. On other hand, very little is known about what is happening at the intermediate levels during virus infection. Here, we use flow cytometry, a technique to rapidly measure large numbers of individual cells, to quantify the number of cells infected by a plant virus, in different leaves and at different times. We found that few cells become infected, and only one or two virus particles typically initiated cellular infection. Moreover, viruses from an infected cell will infect only one or two other cells. Therefore, although viruses replicate at astronomical rates within a cell, their rate of spread between individual cells can be much slower.

## Introduction

For obligate intra-cellular micro-parasites such as viruses, the cell is the fundamental and minimal unit of infection. Important macro-scale phenomena in viral infection – immunity, virulence, transmission, and evolution – all depend on the infection outcome in individual cells. The biochemical and molecular bases of virus infection have received much scrutiny, and in the past decades there also have been major advances in understanding the dynamics at the host and host-population levels. The next great challenge is a unified picture of virus infection dynamics and evolution that integrates different spatiotemporal scales [1], [2]. However, integration across different spatiotemporal scales effectively has not occurred across the between-cell level due to practical and methodological considerations.

At present, there simply is not a coherent picture of infection dynamics at the between-cell level. A number of key issues have not been addressed adequately to date. First, virus replication in an individual cell can be extremely rapid [3], [4], as can the advance of infection and long-range movement [5]. However, little is known about the rate at which infection spreads at the cellular level [6]. What will be the number of newly infected cells per infected cell per day, a value we refer to as the cellular contagion rate (*R*)? Whereas a reproduction ratio estimates the number of cells directly infected by one cell [6], the contagion rate estimates the total number of newly infected cells occurring per infected cell over a given time period. For *Tobacco mosaic virus* (TMV) infection of *Nicotiana benthamiana* plants, a low *R* was estimated (0.5–0.6 cells/cell/d), although why this *R* value was so low was not discussed [7]. Given the rapid replication and spread of viruses, this result is unexpected and it is not at all clear whether other viruses will adhere to similar patterns. Furthermore, a constant *R* value was assumed in the analysis described in ref. [7], whereas a time-varying rate may provide more insights into the underlying dynamics [6]. Another important issue is that individual cells can be observed readily in cell culture systems, whereas gross infection patterns in multi-cellular hosts can be observed by means of virus-induced symptoms, molecular methods [8] or by monitoring infection of tagged viruses [5]. However, these methods do not render information on how the number of infected cells in different tissues changes over time. Finally, variation in genotype frequencies has been described only at higher levels of host organization [9]–[11]. By variation in genotype frequencies, we refer to the differences in the abundance of different virus variants, after a cohort of hosts is initially inoculated with a virus population containing two or more variants. How will this variation change from the population to the individual to the organ, and finally, to the cell? This variation is pivotal to studying the infection dynamics and evolution of viruses. Within-cell interactions between virus genotypes, such as recombination and the complementation of defective virus genotypes, will require that the presence of two genotypes within a host also carry over to the organ and individual cell levels. Whether genotypes carry over will depend on the genetic bottlenecks a virus population passes through when colonizing organs or infecting a cell, respectively.

Plant viruses are ideal model systems for studying virus infection at the between-cell level, and therefore infection dynamics at this level are probably best understood in these systems. The targets of primary infection by mechanical inoculation – epidermal cells – can be readily observed *in situ* [5], [12]–[14], allowing for the tracking of cell-to-cell movement [13]. Moreover, two approaches have been developed to determine whether protoplasts – intact cells extracted after degradation of the cell wall – are infected by different plant virus variants, based on fluorophores [7] or nested PCR [9]. Finally, there is an enviable characteristic of plants: their leaves are natural, biologically relevant compartments that can be removed cleanly (e.g. [15]) for further study.

The development of plant viruses as model systems to study between-cell infection dynamics has led to important insights and the estimation of some key infection parameters. First, as discussed above, a low *R* has been estimated for TMV [7]. Second, estimates of the cellular multiplicity of infection (*MOI*) have been made for three plant viruses. For TMV, *MOI* was found to be low (*MOI*<2) [7], [16]. Moreover, in this particular case a substantial proportion of cells (>0.1) remain uninfected [7]. However, a model-selection-based analysis of the TMV data suggests *MOI* might in fact be higher, whilst the number of coinfected cells is low due to spatial segregation of the two virus variants [17]. For *Cauliflower mosaic virus* (CaMV), *MOI* was reported to vary from 2 to 13 over time, and most cells were infected [9]. Furthermore, for CaMV virion concentrations in vascular tissue are correlated to *MOI* [18]. For *Soil-borne wheat mosaic virus*, *MOI* was estimated during the first rounds of cellular infection in the inoculated leaf, rendering an estimated of 5–6 [12]. Additionally, low level of *potyvirus* cellular coinfections suggest a low *MOI* for *potyviruses* [19]. Finally, for our model system, *Tobacco etch virus* (TEV; genus *Potyvirus*, family *Potyviridae*), the number of infected cells in systemic tissues early in infection depends on the number of primary infection foci, and the number of infected cells does not increase to a frequency greater than 0.5 [15].

Important omissions in our understanding of infection dynamics at the between-cell level remain, however. In particular, a comprehensive view of the between-cell level of infection is missing and the tracking of cell-level infection in multiple host organs or compartments has not been reported. We therefore opted to study these dynamics in TEV and devised an experimental setup in which we could measure infection at the cellular level, which was both sensitive and high-throughput. We opted to analyze the presence of viral variants in individual cells using a flow-cytometry-based method [15], [20]. This approach allows for quantitative measurements of the number of cellular infections for two virus variants in a large number of mesophyll cells, allowing for an analysis of infection dynamics in different host compartments and at different times. This large dataset allowed us to describe the dynamic pattern of the number of infected cells over time, estimate *MOI*, quantify *R*, and consider the variation in genotype frequencies at different levels of host organization as a consequence of bottlenecks.

## Results

### Low levels of cellular infection and coinfection

We generated two TEV variants, TEV-BFP and TEV-Venus, which express blue or yellow fluorescent proteins, respectively. Fluorescent markers inserted in the TEV genome can be stable over multiple short rounds of infection [14], [21], and we confirmed the integrity of the marker sequences throughout the experiment (see Materials and Methods). Furthermore, the insertion of eGFP – a variant of the fluorescent protein from which BFP and Venus are derived – in the TEV genome has no effect on virus accumulation after 7 days post-inoculation (dpi) (see Materials and Methods). Therefore, these marked viruses have biological properties similar to the wild-type virus from which they are derived. We rub-inoculated the third true leaf of *Nicotiana tabacum L.* cv. Xanthi plants with a 1∶1 mixture of infectious saps (ground tissue in inoculation buffer) of the two variants. We then isolated protoplasts [15], [20] from the third, fifth, sixth, and seventh true leaves at 3, 5, 7, and 10 dpi, with five replicate plants for each time point. We did not analyze the fourth true leaf because under the current experimental conditions this leaf does not show any infection. Flow cytometry was used to determine which cells were uninfected, infected by one or by both virus variants. Using this approach we could quantitatively measure the distribution of cellular infection over space and time, for the two virus variants.

**Figure 1:**
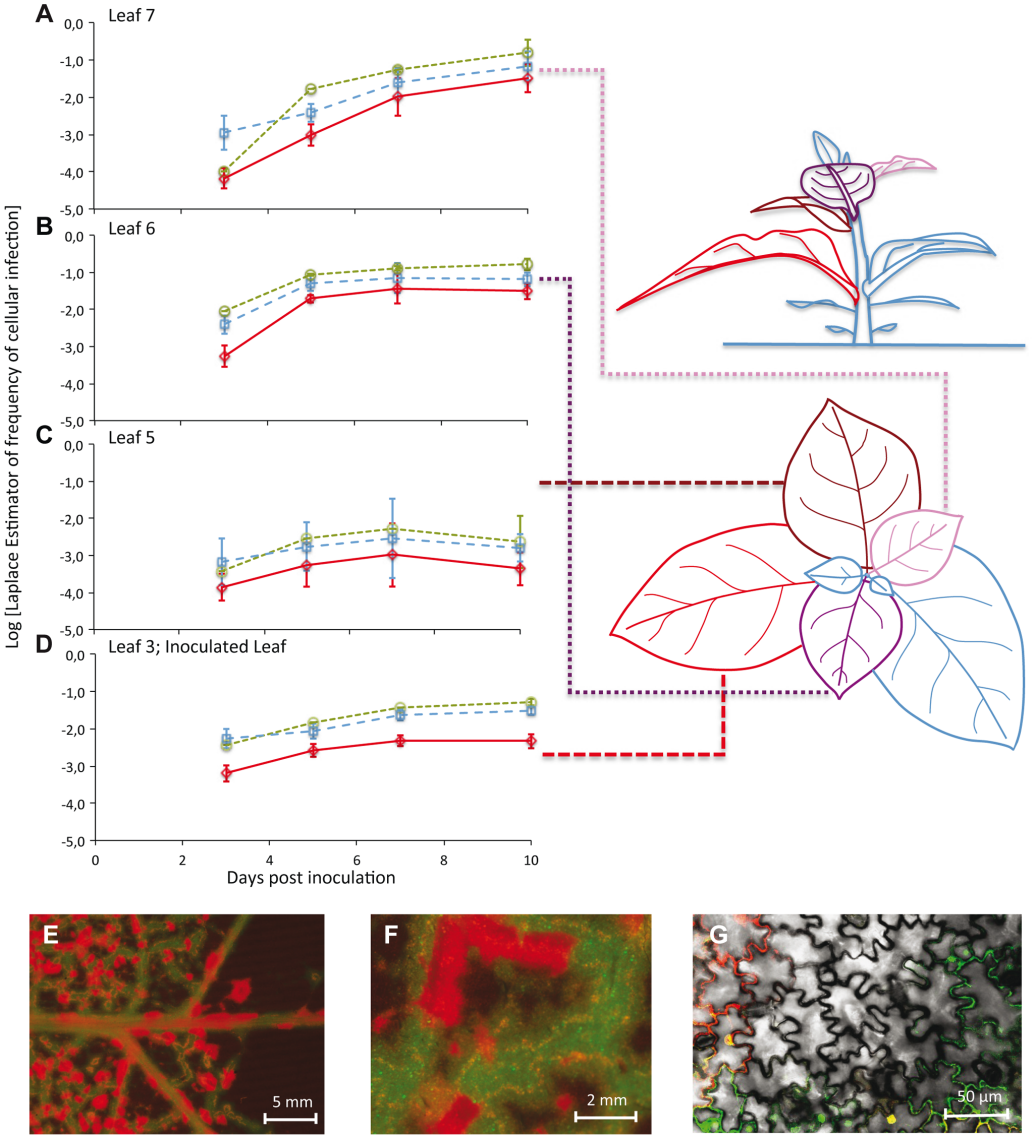
The number of cells infected by each of the two virus variants over space and time. In Panels A–D, the observed frequencies of cellular infection in Leafs 7, 6, 5, and 3 are given for all cells infected by TEV-Venus (finely dotted green line with circles), TEV-BFP (coarsely dotted blue line with squares) and those infected by both variants (continuous red line with diamonds). The abscissae represents days post inoculation (dpi), whilst the ordinate is the frequency at which a particular infected-cell type was observed. Error bars for all panels represent ± 1 SD, and each data point represents the mean of 5 plants, with 50,000 cells measured per individual leaf. Leaf 4 was not included in this analysis because it does not become infected, probably because the host vasculature does not transport virions to this leaf (see Discussion). The leaves analyzed in this study have been given different colors in the schematic representation of the plant for the sake of easy identification in the top and side views. Note that for the side of view of the tobacco plant, stem length has not been drawn to scale – being shorter than depicted here – for the sake of clarity. In panels E–F, stereomicroscopic images of Leaf 6 at 7 dpi of a plant inoculated with TEV-eGFP (green fluorescence) and TEV-mCherry (red fluorescence) are shown. Entire regions of leaf remain uninfected even though cell infection has saturated, probably because a phloem-transported virus cannot traverse the sink-source boundary. However, even prior to the boundary (panel F) there remain uninfected regions. Panel G shows confocal microscopy of a region that appears to be completely infected at higher scales (panels E and F), being a merge of the trans, eGFP and mCherry images.

The frequency of virus-infected cells was low (mean ± 1 SD: 0.072±0.099), with the highest level of infection observed in any one sample being 0.424 (Leaf 7 at 10 dpi) (Figure 1A–D). The frequency of cells infected by both virus variants was also low (mean ± 1 SD: 0.012±0.023), with the highest level of coinfection observed in any sample being 0.112 (Leaf 6 at 7 dpi) (Figure 1A–D). These low levels of coinfection are in agreement with previous studies on plant RNA viruses [7], [13], [19], and suggest that *MOI* is low. Few cells were infected in any leaf at 3 dpi, with the greatest number of infections being found in Leaves 3 and 6. This surprising observation can be explained by the occurrence of limited, relatively slow TEV expansion at the macroscopic level in the inoculated leaf [8], combined with fast egress (<2 dpi) from Leaf 3 to Leaf 6 at high viral doses [15]. Both infection and coinfection appear to increase over time in the different leaves, although Leaf 5 shows very low levels of infection. Infection progresses slower in Leaf 3 than in Leaves 6 and 7. Leaf 6 becomes infected before Leaf 7, but the dynamics in these two leaves are otherwise very similar. The frequency of TEV-Venus infected cells was significantly higher than expected for a 1∶1 inoculum (one-sample *t*-test: *t*_79_ = 4.141, *P*<0.001), although the magnitude of the deviation was small (mean Laplace point estimator for the frequency of TEV-Venus infected cells ± 1 SD = 0.591±0.196). This deviation could occur because of a small discrepancy in the inoculum ratio, or a small difference in infectivity or in within-host competitive fitness of the two variants. To confirm that infection levels in Leaf 7 had saturated at 10 dpi, in a separate experiment we also analyzed infection in Leaf 7 at 13 dpi. The observed frequency of virus-infected cells was slightly lower than at 10 dpi, although the difference was not statistically significant (two-sample *t*-test: *t*_8_ = 1.251, *P* = 0.246). The data therefore suggest that infection levels had saturated in all analyzed leaves by 10 dpi.

To visually illustrate patterns of infection, we infected plants with TEV-eGFP and TEV-mCherry [14] under identical conditions. These viruses were used here, instead of TEV-BFP and TEV-Venus, because their fluorescent proteins are more suitable for microscopy. Even when infection appears to have saturated at both the cell and visible fluorescence level, we could see heterogeneities in the distribution of virus infection over the leaf at different spatial levels (Figure 1E–G).

### Cellular contagion rate

**Figure 2:**
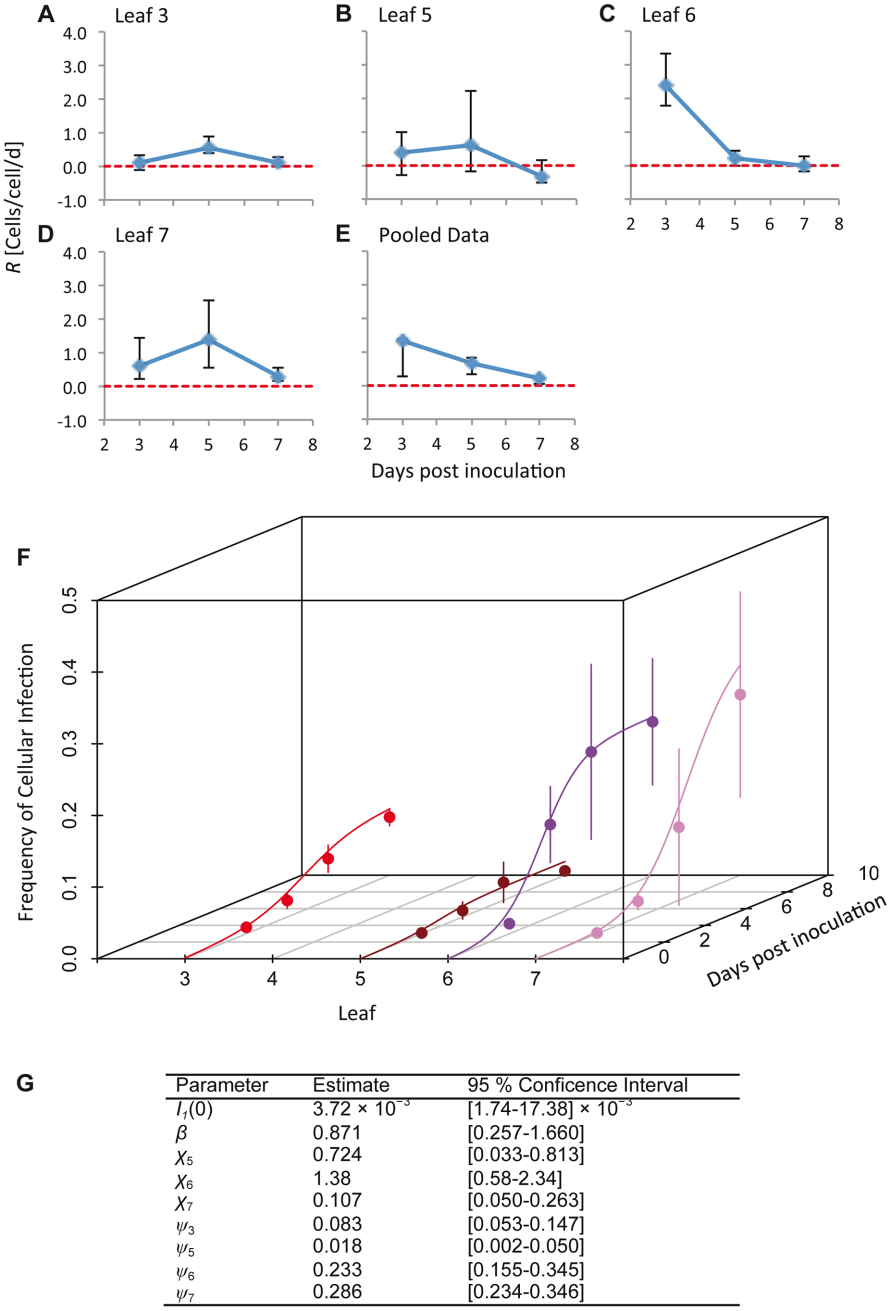
The estimated time-varying cellular contagion rate (*R*) and results for the model of within-host spread. In panels A–D, estimates of *R* (ordinate) are shown for different days (abscissae) and leaves, with error bars representing the 95% CI. Note that although the estimates for Leaf 5 vary appreciably over time, the 95% CIs overlap with each other and with zero, meaning there is no evidence for the appreciable spread of cellular infection in this leaf. For all panels each data point represents 5 plants. Panel E shows *R* estimates for the pooled data. In panel F, the frequency of infected cells at different times in Leaves 3 (inoculated leaf), 5, 6 and 7 is shown. The lines are the fitted Susceptible-Infectious (SI) meta-population model, whilst the circles are the data points and error bars ± 1 SD. In Panel G, we provide estimated model parameters for the SI model and their 95% CI.

We estimated the time-varying cellular contagion rate (*R*) from the data using a simple maximum likelihood method. This analysis was carried out on the total number of infected cells, regardless the virus variants present. For *R*>0 the number of infected cells increases, whereas for *R*<0 it decreases. Our estimates of *R* for individual leaves (Figure 2A–D) ranged from 2.43 cells/cell/d (95% CI: 1.80–3.39) (Leaf 6, 3 dpi;) to values not significantly different from zero (e.g., −0.327 cells/cell/d (95% CI: −0.539–0.271) for Leaf 5, 7 dpi). We do not expect *R*<0 in this system, since infection is not cleared and the number of infected cells can therefore not decrease. Our approach might slightly overestimate *R* in individual leaves because of between-leaf transmission, and we therefore also estimated *R* for pooled data from different leaves (Figure 2E). One disadvantage of this approach is that tissues with high infection levels will most strongly affect *R* estimates. These estimates of *R* (mean [95% CI]) ranged from 1.342 cells/cell/d [0.247–1.371], 3 dpi, to 0.196 cells/cell/d [0.041–0.244], 7 dpi, and were always significantly greater than zero. Overall, values of *R* appear to be surprisingly low given estimates of the rapid rate of cell-to-cell movement for TEV during initial infection, whilst they are similar to estimates of *R* for TMV (0.5–0.6 cells/cell/d) [7]. Low *R* values may therefore be commonplace in plant RNA viruses, although data from more pathosystems will be needed to confirm this idea.

Dolja et al. [5] observed that a primary infection focus starts with a single infected cell and grows to formation with a diameter of ten infected cells within 24 h, and hence 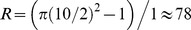 cells/cell/d. This calculation is conservative and underestimates *R* because infection in the first infected cell cannot be observed at *t* = 0, and because it only takes into account infection in the epidermal cells. Note that such a high value – which probably far exceeds the number of other cells to which each cell is plasmodesmally connected [7] – is possible because of multiple rounds of replication can occur within a single day [5]. The *R* values we have measured are therefore extremely low compared to *R* values found in the inoculated leaf during early infection.

### Within-host viral spread

We wanted to test whether our understanding of the process that is likely to govern cell-level infection patterns was congruent with our empirical data. Specifically, we wanted to test whether there were leaf-dependent differences in key infection parameters, and whether there was evidence for aggregation of virus-infected cells limiting infection spread. We therefore developed a simple susceptible-infectious (SI) model of within-host infection dynamics. Each leaf in a plant represents a physically separated compartment - with its own physiological state - that a virus must colonize [22]. We therefore developed a simple meta-population dynamics model with between-leaf transmission from lower leaves to upper leaves. For the *k* ^th^ leaf, the rate of change of the fraction of infected cells (*I_k_*) is:
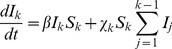
(1)where *β* is the within-leaf transmission coefficient (from cell to cell), *χ* is the between-leaf transmission coefficient and *S* is the fraction of susceptible cells. Between-leaf transmission depends on the total fraction of infected cells in the leaves below the *k* ^th^ leaf, given that systemic-movement for phloem-transported viruses is towards the apical sink leaves [5], [22]. Potyvirus infection appears to be marked by the aggregation of infected cells [19], and given that plant cells will largely retain their respective positions in developed leaves, the perfect mixing assumptions of the SI model will not be met. We therefore included a spatial aggregation factor of infectious units (*i.e.*, infected cells) *ψ_k_* in the model, such that
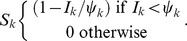
By spatial aggregation of infected cells, we mean that infected cells are likely to be found together and are therefore not randomly distributed in the leaf. The mechanism resulting in the spatial aggregation of infected cells is probably the dependence of plant viruses on cell-to-cell movement for local infection to spread: the spread of virions, or in some cases unencapsidated genomes, from an infected cell to its direct neighbors [5], [13]. When *ψ_k_* = 1 there is perfect mixing, whereas when *ψ_k_* approaches 0 there is maximum aggregation of infected cells [23], [24]. The model was fitted using maximum likelihood methods, and model selection was performed to ensure the data supported the inclusion of all model parameters (see Materials and Methods). As with the estimates of *R*, this analysis was carried out on the total number of infected cells and does not distinguish between the two virus variants.

The SI meta-population model could describe the data well, clearly capturing the main trends in the data (Figure 2F). Spatial aggregation of infected cells (*ψ_k_*) was indispensable to the model (Table S2), and parameter estimates varied over leaves; *ψ_k_* was most pronounced in Leaves 3 and 5, and much lower in Leaves 6 and 7 (Figure 2G). The between-leaf transmission coefficients (*χ_k_*) for Leaves 5 and 6 were similar, although infection never reaches even moderate levels in Leaf 5. *χ_7_* was significantly lower than *χ_6_* (non-overlapping 95% CIs of parameter estimates), although the number of infected cells in both leaves reached moderate levels eventually. Parameter estimates therefore suggest that infection dynamics vary for each leaf, even though the overall pattern (Figure 1A–B) is similar for Leaves 6 and 7.

### Cellular *MOI*

**Figure 3:**
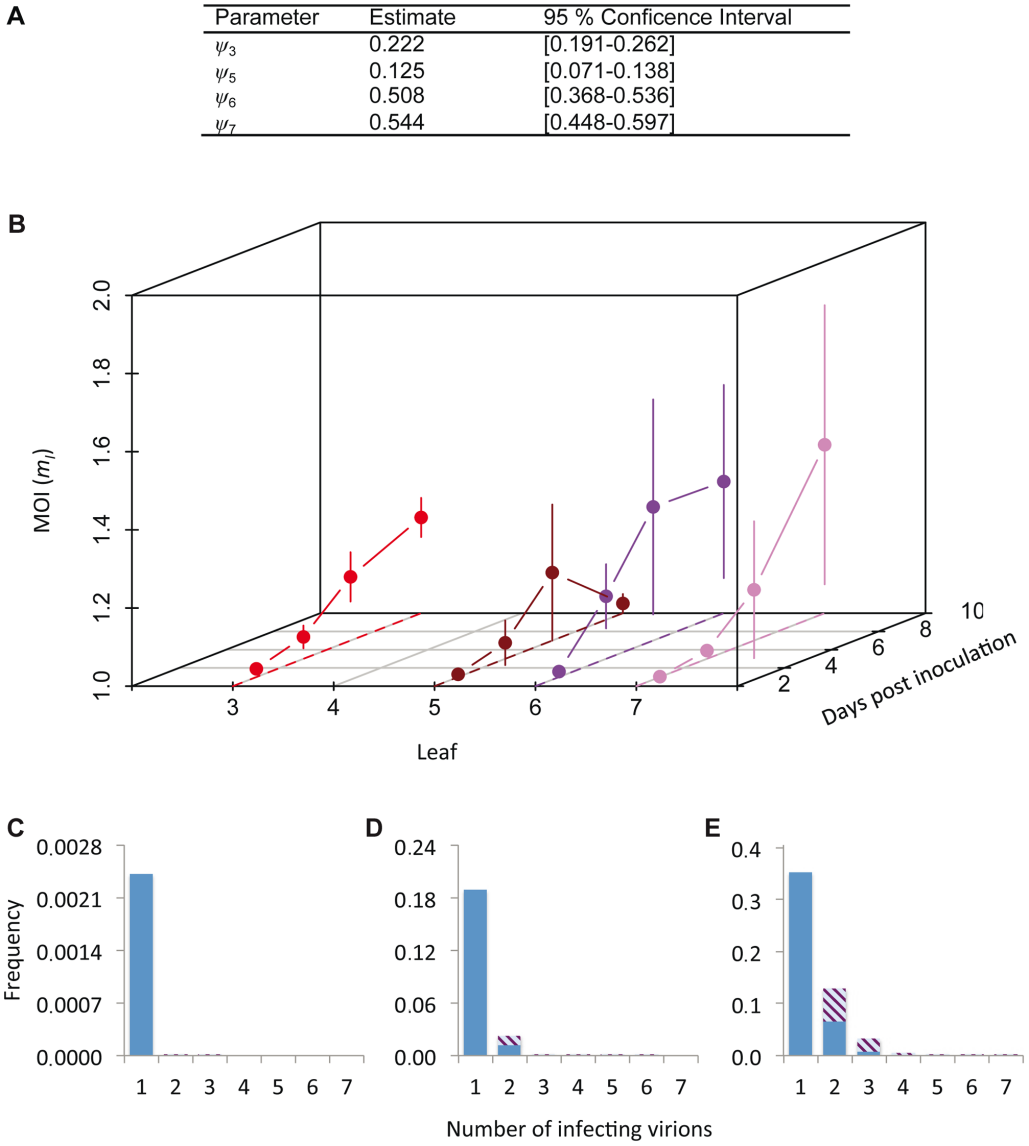
Cellular *MOI*. The results of *MOI* model fitting and *MOI* estimates are given. In panel A, we provide estimates of *ψ*, the leaf-dependent infection aggregation parameter, for the best-supported model. In panel B, estimates of cellular *MOI*, for different times post-inoculation and in different leaves are given. Error bars represent the 95% confidence interval. Note that the reported *MOI* value is *m_I_*, the *MOI* in infected cells only, which has a minimum value of 1. *MOI* is initially very low and gradually increases, never reaching 1.5. *MOI* values for the final time point (10 dpi) are similar for Leaves 3, 6 and 7, whilst it remains very low for Leaf 5, which hardly becomes infected. Panels C–E show model predictions for the frequency at which cells are infected by a certain number of virions. The blue section of the bar indicates the frequency of infection by only one virus variant, whereas striped area indicates coinfection by both virus variants, assuming a 1∶1 ratio of virus variants. Panel C gives this prediction for the lowest *MOI* (*m_I_* = 1.001), panel D for the mean *MOI* (*m_I_* = 1.137), and panel E for the highest *MOI* (*m_I_* = 1.432). Estimated *MOI* values are low, but the number of infecting virions is assumed to follow a Poisson distribution. Hence even at the low mean *MOI* some cells will be infected by 2 or more virions, allowing for cellular coinfection.

The cellular *MOI* can be estimated from our data, as has been previously done for two plant viruses with a similar experimental setup [7], [9]. However, estimates of *MOI* can be influenced by the estimation method [17]. Model selection was therefore performed on a set of nine *MOI*-predicting models (see Materials and Methods), by testing which Poisson-based model best predicted the relationship between the fractions of uninfected and coinfected cells (i.e., those cells infected by both virus variants) [17]. The models incorporated spatial segregation of virus genotypes, spatial aggregation of infected cells, superinfection exclusion at the cellular level and combinations of all these effects. We could thereby identify the best model to generate *MOI* estimates (Tables S3 and S4). The best-supported model incorporated only a leaf-dependent aggregation factor *ψ* (Table S4). The *MOI* and SI model selection results are therefore in good agreement with each other, although estimated *ψ* values were higher than those obtained from the SI model (Figure 2G), indicating less aggregation (Figure 3A). These two separate model selection procedures therefore confirm the importance of the spatial aggregation of infected cells for understanding TEV infection dynamics at the between-cell level, as might be expected for a virus that spreads by cell-to-cell movement. On the other hand, in a similar model-selection-based analysis for TMV and CaMV *MOI*, two viruses that also move by cell-to-cell movement, spatial aggregation only marginally improved model fit for both datasets [17]. These two different model-selection results suggest that whether cell-to-cell movement really has an impact on *MOI* estimation will depend not only on the mechanism of movement. Other factors, such as the number and distribution of initially infected cells, and the frequency of infected cells, also may play an important role.

We then derived predictions of *MOI* using the best-supported model (Figure 3B). As could be expected from the low frequencies of cellular infection and coinfection (Figure 1A–D), the predicted *MOI*s were low, ranging from 1.001 (Leaf 5, 3 dpi) to 1.432 (Leaf 6, 7 dpi). Note that we report the estimated *MOI* value in infected cells only (i.e., *m_I_* in Materials and Methods), which has a minimum value of 1. The corresponding range of *MOI* values calculated over the whole population of infected and uninfected cells (*m_T_*) is 0.002 (Leaf 5, 3 dpi) to 0.735 (Leaf 6, 7 dpi). Although these estimates may seem low intuitively, *MOI* is assumed to follow a Poisson distribution over cells and some cells can still be infected by two or more virions, even when the mean of the distribution is low (Figure 3C–E). Our estimates of MOI are similar to the low estimates for TMV [7], [16], although model-selection-based estimates for the TMV data result in *MOI* values ranging to somewhat higher values (up to 2.1), due to the predicted occurrence of spatial segregation of virus genotypes [17]. For CaMV much higher *MOI* values were observed later in infection [9], but in our system infection levels remain low even then.

### Variation in genotype frequencies at the host, leaf and individual-cell levels

**Figure 4:**
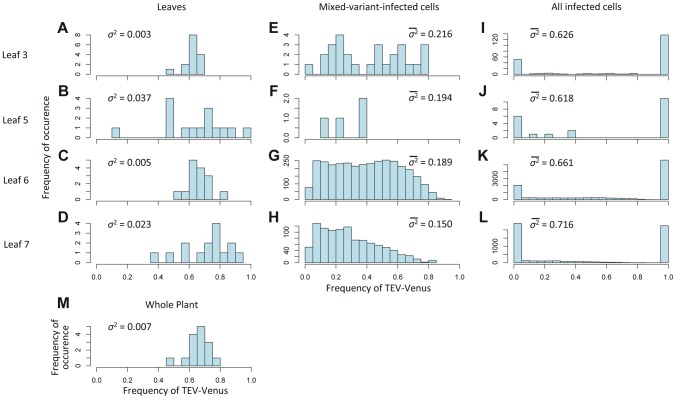
The variation in genotypic frequencies at the host, organ and individual-cell levels. In Histograms of TEV-Venus frequency at the level of leaves (A–D), cells coinfected by both virus variants (E–H), all infected cells (I–L) and finally for the whole plant (M) are given. For the leaf and whole-plant data (A–D and M), all the data from days 5, 7 and 10 were pooled and the variance (*σ* ^2^) is given. For the cellular data (E–L), one leaf from a given leaf level was randomly selected (from all replicates at days 5, 7 and 10) for display in the histogram, and the mean variance (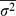) over all replicates is given. The data demonstrate that although the variation in genotype frequencies at the level of the whole plant and leaves is limited, the variation at the individual cell level is much higher.

**Figure 5:**
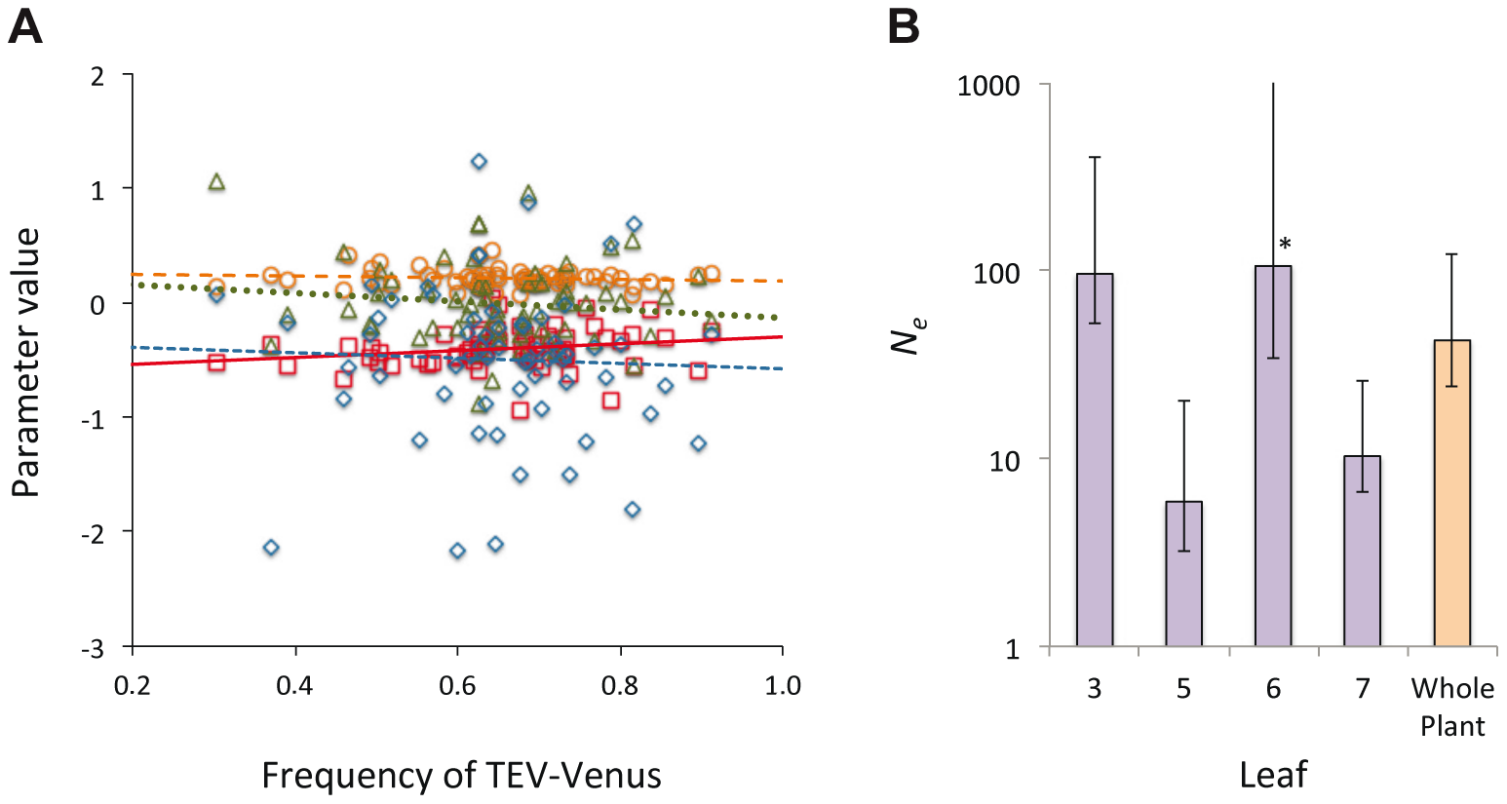
The distribution of genotypic frequencies within cells and estimates of effective population size (*N_e_*). In panel A, the distribution of the log-transformed TEV-Venus∶TEV-TagBFP ratio in cells infected by both variants is shown, using the combined data from days 3–10. On the abscissae is frequency of TEV-Venus in the whole leaf and on the ordinate is the value for parameters describing the distribution of the log-transformed virus ratio, with individual points representing the data and lines representing ordinary least squares regression lines. Red squares and solid line correspond to the mean, orange circles and the coarse dotted line are the variance, blue diamonds and the intermediate-grain dotted line are the kurtosis, and green triangles and fine dotted line are the skewness. There was not a significant relationship between TEV-Venus frequency and any of the distribution parameters (Model 2 regression), suggesting that the virus genotype ratio in individual coinfected cells is largely independent of infection events at the leaf level. In Panel B, we estimated *N_e_* for each leaf and the whole plant, and the estimates with 95% confidence intervals from the pooled data of days 5, 7 and 10 are given (each data point represents 15 plants). *N_e_* for the inoculated leaf was about 100, which corresponds well with the approximate number of primary infection foci. There were considerably smaller *N_e_* values for Leaves 5 and 7. For Leaf 6, the estimated bottleneck size was about the same for the inoculated leaf, although the 95% extends to ∞ (marked by an *). *N_e_* estimates therefore suggest the virus populations infecting different leaves vary in size, although *N_e_* estimates are not entirely congruent with estimates of between-leaf transmission (Figure 2B) at first glance.

The experimental data also allow us to consider variation in the frequencies of viral genotypes at different levels of the host: leaf (Figure 4A–D), cells coinfected by both virus variants (Figure 4E–H), all infected cells (Figure 4I–L), but also at the level of the host-plant population (Figure 4M). Variance of TEV-Venus frequencies appears to increase strongly from the plant and leaf levels to the individual cell level (Figure 4A–M). The log-transformed genotype ratios (TEV-Venus∶TEV-BFP) in individual cells appear to be independent of the frequency of TEV-Venus in the leaf (Figure 5A), indicating a decoupling of processes occurring at the leaf and coinfected-cell levels. Low estimates of *MOI* (Figure 3B) imply that the virus population entering each cell is subject to a narrow genetic bottleneck. A decoupling of the infection processes at the leaf and cell levels is predicted to occur because very few cells are infected by more than 2 virions (Figure 3E). Hence, for the vast majority of coinfected cells the frequency of virus variants, as represented by the infecting virions, is limited to 1/3, 1/2 and 2/3. If our *MOI* estimates are correct, than stochasticity in the replication process within the cell accounts for high levels of variation. In line with these expectations, we observed high levels of variation in virus variants at the cellular level (Figure 4E–H) and a distribution of variants in coinfected cells that is independent of the frequency of virus variants in the leaf (Figure 5A). Note that there are couplings between the leaf and cell-level dynamics (i.e., *MOI* depends on the overall level of infection for the best-supported *MOI* models; see Materials and Methods), but our observations show that not all leaf-level characteristics of the virus population carry over to individual cells.

Finally, we estimated the effective population size, *N_e_*, for individual leaves and the whole plant [25] (see Materials and Methods). For the inoculated leaf we obtained a *N_e_* estimate of approximately 100 (Figure 5B), corresponding well to the approximate number of primary infection foci observed. For Leaf 6, *N_e_* was also estimated to be approximately 100, although the confidence interval extends to ∞ and there is no evidence for a genetic bottleneck in this leaf. For Leaves 5 and 7, much lower estimates of *N_e_* were obtained, suggesting that fewer virions infect these leaves and that it is more difficult for the virus to invade these compartments. A wide range of within-host effective population sizes at the leaf level has been reported for different viruses [10], [11], [18], [26]. Here we show a similar range of effective population sizes can occur with a single virus-host combination, probably due to the combined effects of host physiology, anatomy and immunity.

## Discussion

To link infection dynamics at the cell and host levels, we have measured the number of cells infected by two virus variants within individual plants over time and space. We have estimated *R* (the cellular contagion rate, expressed as newly infected cells per infected cell per day) over time for systemic virus infection. A conservative estimate of the maximum value for *R* is 1.4 cells/cell/d on day 3 (Figure 2E), and it falls to just under 0.2 cells/cell/d by day seven. These values are comparable to estimates of *R* for TMV infection of *N. benthamiana* of 0.5–0.6 cells/cell/day, although in this instance a constant *R* was estimated [7]. We can therefore conclude that for our model system, and perhaps more generally for plant RNA viruses, *R* is very low during systemic infection, suggesting that most cells will transmit virus to one or possibly even zero other cells during infection.

Here we have estimated the cellular contagion rate over a period of one day. Given that TEV infection has been reported to expand at a rate of one row of cells every 2 h [5], it is entirely possible that multiple rounds of infection will occur during one day. Therefore, the reproduction ratio at the cellular level (i.e., the number of cells to which one infected cell spreads infection over its lifetime) is probably similar to, or even lower than our estimates for the cellular contagion rate. These estimates are in principle the aggregated effect of local cell-to-cell movement and long-range systemic movement. What then accounts for these surprisingly low values of *R*, and are they reconcilable with high replication rates at the molecular level [3], [4] and fast virus expansion throughout the plant [5], [15]?

Decreases in cellular replication because the carrying capacity for infection has been reached do not explain these observations: low *R* values were estimated when infection levels were very low (e.g., compare Figures 2C and 2F). However, contagion rates at the cellular level can be much higher than those we have observed here: based on other results [5] we also estimate that during expansion in primary infection foci *R*≈78 cells/cell/d. We have observed early infection in systemically infected leaves that eventually reached high levels (i.e., Leaves 6 and 7), and especially in the case of Leaf 7 these infections appears to be initiated by a small number of virions. Hence, *ceteris paribus* we would have expected high *R* levels in these leaves as well, and moreover in Leaf 7 *R* does not reach the same levels as Leaf 6. These observations implicate two processes in slowing the observed rate of virus expansion at the between-cell level. First, host immune responses, particularly RNA silencing [27], is very likely to play a role. Moreover, since a specific RNA silencing signal progresses systemically to sink leaves [27], [28], we speculate that this may explain why there appear to be lower *R* levels in Leaf 7 than in Leaf 6. Second, our experimental approach limits us to analyzing the cells in a leaf as a whole, whereas the analysis of cells in the infection front would result in higher *R* values.

We found striking differences in infection dynamics in different leaves (Figure 1A–D). These differences were also reflected in estimates of parameters for the different models fitted to the data (Figures 2B, 3A and 5B). What can account for the infection dynamics in different leaves? First, sink-source transitions will play a major role in determining if and to what extent leaves can be colonized, because phloem-transported viruses cannot cross the sink-source boundary in any leaf [22]. This functional boundary separates the basal part of a developing leaf, which is importing photo assimilates, from the distal part that is already exporting them. Furthermore, sink-source transitions may further impact the spatial aggregation of infected cells on a smaller spatial level: sink-source transitions will determine from which classes of phloem the virus can unload, with much less restriction in smaller veins prior to the transition [22]. Hence the distribution of initially infected cells is likely to be more homogeneous – also on small spatial scale – in sink leaves, leading to less spatial aggregation of infected cells. We saw infection only in the basipetal region of Leaf 5, whereas about half of the surface of Leaf 6 became infected (Figure 1E). Therefore, we think that Leaf 4 has probably completed the sink-source transition, and is almost exclusively exporting photo assimilates, whereas it has not affected much of Leaf 7. These assertions on the physiological state of these different leafs are strongly supported by measurements of polyamine levels [29], which are molecular markers of proliferating source tissues. Putrescine and spermidine levels show that for *N. tabacum* cv. Xanthi of the same development stage as our plants at inoculation, the sink-source transition is virtually complete in Leaf 4, almost complete in Leaf 5, and has not yet commenced in Leaf 6. Note that whereas sink-source transitions probably account for virus aggregation on a large and intermediate scale (Figure 1E–1F), RNA silencing probably impedes infection at all scales, also resulting in aggregation of infected cells on the smallest scales (Figure 1G) [27]. Second, crossing from leaves at one side of the plant to the opposite can be hindered by the phloem connections between leaves [22]. Third, as aggregation of infected cells is increased, the rate of virus spread decreases [23] and the plant will have more time to mount an effective response [27]. Consequently, we hypothesize that large effective population sizes can only be achieved if (i) the virus can be readily transmitted between two particular leaves, and (ii) the subsequent aggregation of infected cells is moderate to low (e.g., the virus is not restricted to the basal part of the leaf by the sink-source transition), allowing infection to expand beyond the initial point of entry.

Based on these other studies, we therefore speculate on what processes can account for the leaf-dependent differences we have observed. Infection progresses relatively slow in Leaf 3, probably because under the conditions used the virus only expands locally and egresses from this source leaf [8]. Leaf 4 probably never shows any infection in our setup because it has completed the sink-source transition, and is moreover located opposite Leaf 3 (for leaf positions see Figure 1). Leaf 5 has a relatively high between-leaf transmission, strong aggregation, and a small bottleneck size (Figures 2, 3 and 5). Its position directly above the inoculated leaf explains high between-leaves transmission, whilst the nearly complete sink-source transition results in high aggregation, low levels of infection and therefore a *de facto* genetic bottleneck. In line with this explanation, the highest levels of aggregation were observed in Leaf 5, suggesting the virus expansion is very constrained in this leaf. Leaf 6 has a high between-leaf transmission due to its position above the inoculated leaf. Moreover, because the sink-source transition is far from complete there are high levels of infection, moderate aggregation and no genetic bottleneck. Finally, Leaf 7 is positioned on the far side of the plant, with respect to the inoculated leaf, and the increasing intensity of host immune responses results in low between-leaf transmission, and hence a genetic bottleneck occurs. However, since the sink-source transition is far from complete, those viruses that do enter the leaf can expand prolifically, resulting in lower estimated levels of aggregation and high infection levels. In summary, we think that plant anatomy and physiology may largely explain the leaf-dependent differences in infection patterns we have observed, although our explanation will require further testing.

Our analyses of infection spread and *MOI* support the idea that aggregation of virus-infected cells is also important for understanding dynamical patterns and therefore low *R* values. If there is aggregation of virus-infected cells, which is concurrent with potyviruses achieving local spread by cell-to-cell movement, only those cells on the edge of an aggregate can contribute to virus expansion, and even fortuitously situated cells may not actually infect those susceptible cells they are in contact with before neighboring cells do. The limitations on virus spread from an individual cell to its neighboring cells due to the overall rapid spatial spread of the virus is an effect we refer to as “self-shading”. The importance of self-shading in limiting between-hosts spread [23], [30], and its implications for virulence evolution [31], [32], have been recognized on larger spatial scales. Our results stress the importance of extending these concepts to within-host dynamics, although we anticipate that there will be differences in the between-host and within-host levels. For example, we hypothesize that a high cellular contagion rate may not incur a major cost in our model system; host cells are static and once a tissue has been infected there are no possibilities for further within-host spread, except for phloem loading in a minority of cells. Therefore, we speculate that aggregation and self-shading will, in this case, impose selection for fast viral replication and spread at the within-host level.

Our experimental approach consisted of the isolation of protoplasts, followed by measurements on individual cells by flow-cytometry. Advantages of this approach are its amenability to high-throughput, the high sensitivity of the flow-cytometer, and the fact that mesophyll cells – the primary targets of virus replication – can be analyzed. Disadvantages are the fact that sampling is destructive, and hence a time course cannot be analyzed, and the spatial information is lost during protoplast extraction. Compared to other techniques available for analyzing protoplasts [7], [9], the approach used here has a much higher throughput. Our approach may have a higher sensitivity than microscopy [7], although PCR-based methods are probably more sensitive [9]. Another alternative approach to analyze virus infection dynamics would have been microscopy on whole leaves, which renders spatial information and allows for longitudinal analyses [13]. Although this approach works very well in the inoculated leaf [13], it is not clear how well it would function in systemic leaves, and this is also a lower-throughput method. For a comprehensive analysis such as we have presented, the high-throughput nature of the assay is essential and dictated our choice of experimental approach.

For many other virus-host pathosystems, including those that result in disease in animals such as humans, important spatial characteristics of virus-plant pathosystems may be absent. Short-range virus infections can typically be achieved by diffusion of virions instead of cell-to-cell movement, and most host organs will not have the planar anatomy of leaves. However, there are general characteristics of virus-host interactions that suggest infection aggregation may be a very commonplace phenomenon. First, there are many physical barriers to virus expansion, structuring the host environment and naturally favoring aggregation of infected cells. Second, many viruses replicate in a limited number of cell types or tissues, thus leading to spatial aggregation. Third, epithelia are often targets of viral entry and one of the sites of replication, and consist of highly planar structures. Finally, even for free virions, diffusion and virion removal rates will determine at what distance infection tends to spread. Based on our results and these general considerations, we therefore speculate that aggregation of virus-infected cells and self-shading are likely to be key ingredients for cell-level infection dynamics in a broad range of intra-cellular pathogens infecting complex, multi-cellular hosts.

## Materials and Methods

### Experimental procedures

#### Construction of TEV-Venus and TEV-BFP

An infectious plasmid containing the TEV genome (GenBank DQ986288) [33] was used to construct the TEV-Venus and TEV-BFP genotypes in which these two fluorescent marker genes were inserted between TEV P1 and HC-Pro cistrons. Venus [34] or TagBFP [35] cDNA was amplified by PCR using primers forward 5′-ATGGTGAGCAAGGGCGAGGAG-3′ and reverse 5′-TTGGAAGTACAAGTTTTCTCCGCCCTTGTACAGCTCGTCCATGC-3′ (Venus), or forward 5′-ATGAGCGAGCTGATTAAGGAG -3′ and reverse 5′-TTGGAAGTACAAGTTTTCTCCGCCATTAAGCTTGTGCCCCAGTTTG-3′ (TagBFP). The reverse primers inserted two glycines, as spacers, and a partial NIa-Pro proteolytic site downstream of Venus or TagBFP sequences. This partial proteolytic site (ENLYFQ) is complemented by the first contiguous serine in HC-Pro cistron to mediate marker release from the viral polyprotein. The pTV1a vector, which contains the first 3221 nt of the TEV genome including the complete P1 to HC-Pro cistrons, was amplified using the forward primer 5′-AGCGACAAATCAATCTCTGAGGC-3′ and reverse primer 5′-TTTGTCGCTATAATGTGTCATTGAG-3′. The PCR-amplified Venus and TagBFP sequences were then ligated into the amplified vector sequence, and transformed into electrocompetent *Escherichia coli* DH5α. The identity of the resulting pTV1a-Venus and pTV1a-BFP plasmids was checked by restriction digests and sequencing. Finally, *Pau*I-*Aat*II restriction fragments from pTV1a-Venus and pTV1a-BFP were ligated into *Pau*I-*Aat*II digested pMTEV to construct pTEV-Venus and pTEV-BFP. All PCR reactions were performed with high fidelity Phusion DNA polymerase (Finnzymes).

#### *In vitro* RNA transcription and inoculation

TEV-Venus and TEV-BFP infectious plasmids were linearized with *Bgl*II (TaKaRa) and transcribed into 5′-capped RNAs using the SP6 mMESSAGE mMACHINE kit (Ambion Inc). Transcripts were precipitated (1.5 vol of DEPC-treated water, 1.5 vol of 7.5 M LiCl, 50 mM EDTA), collected and resuspended in DEPC-treated water [36]. Four-week-old *N. tabacum* plants were mechanically inoculated on the third true leaf with RNA TEV transcripts mixes (10 µg). Plants were maintained in the green house at 25°C and 16 h light for one week. Infected tissues were collected 7 dpi. Stocks of infectious virions obtained from freshly TEV-Venus and TEV-BFP infected *N. tabacum* were used as source of TEV inoculum for our experiments.

Concentrated saps of TEV-Venus and TEV-BFP were then obtained by grinding 500 mg of infected tissue in a mortar with 800 µl of inoculation buffer (50 mM potassium phosphate, 3% PEG6000, pH 7.0). Viruses were inoculated separately, or by a 1∶1 mixture of infectious saps on five-weeks-old *N. tabacum* plants. Inoculation was performed by abrasion of the third true leaf with 15 µl of each infectious sap.

#### Test of marker sequence integrity

To test whether the marker sequences were stable throughout the experiment, we performed a test similar to that described in [14]. RNA was extracted 12 dpi from plants infected with TEV-Venus and TEV-BFP, RT was performed (primer: 5′-CGCACTACATAGGAGAATTAG-3′), and finally PCR with primers flanking the marker gene (primers: 5′- CAATTGTTCGCAAGTGTGC-3′ and 5′-ATGGTATGAAGAATGCCTC-3′). Only PCR products corresponding to the virus with the intact marker sequence were found, and not shorter PCR products associated with deletions in the marker gene.

#### Microscopy

To verify the equal proportion of both genotypes in the mix of TEV-Venus and TEV-BFP, fluorescence was observed 3 dpi with a Leica MZ16F stereomicroscope, using a 1× objective lens, GFP2 filter (Leica Microsystems Heidelberg GmbH) for TEV-Venus and Violet filter (Leica) for TEV-BFP to count foci of primary infection on the inoculated leaf. Plants showed equal levels of primary infection for both viruses. To observe TEV-eGFP and TEV-mCherry, using a 0.5× objective lens and GFP2 and dsRed filters (Leica), respectively. Infected plant tissues were observed with a Leica TCS SL spectral confocal microscope using an oil immersed HCX PL APO ×40/1.25-0.75 CS objective (Leica). eGFP-derived fluorescence was observed by excitation at 488 nm from an argon laser and emission at 500–520 nm, whereas mCherry was observed by excitation at 543 nm from a green-neon laser and emission at 600–620 nm. Fluorescence profiles were analyzed and merged using the Leica Confocal Software (version 2.61 build 1537; Leica).

#### Accumulation of TEV and TEV-eGFP

To test whether eGFP-based marker genes had an important effect on the biological characteristics of the virus, *N. tabacum* plants were infected with and equivalent doses of virions of TEV or TEV-eGFP [14]. Virions were quantified by means of a quantitative real-time RT-PCR (RT-qPCR), using PrimeScript RT-PCR kit II (TaKaRa), on the coat protein (CP) (primers: 5′-TTGGTCTTGATGGCAACGTG-3′ and 5′-TGTGCCGTTCAGTGTCTTCCT-3′). A Prism 7500 sequence analyzer (Applied Biosystems) was used, as well as Prism 7500 software, version 2.0.4 (Applied Biosystems), to analyze the data. All aerial plant tissue except the inoculated leaf were collected 7 dpi, RNA was extracted, and a second RT-qPCR using CP primers was performed to determine accumulation. There was not a significant difference in accumulation levels between TEV and TEV-eGFP (*t*-test on log_10_-transformed data: *t*_14_ = 0.754, *P* = 0.463). Therefore, biological properties of the marked virus are similar to those of the wild-type virus. On the other hand, the insertion of marker proteins does appear to affect viral within-host competitive fitness [21], [37].

#### Protoplasts extraction and fluorescence analysis by flow cytometry

Protoplast were extracted using a modification of previously published protocols [20], where sliced leaves were incubated with enzymatic solution (4.3 g/l MS salts, 0.04% cellulase, 0.015% pectinase, 0.6 M manitol, pH 5.8) in dark at 22±2°C. The incubated solution containing protoplast was filtered and centrifuged (4 min, 700 rpm). Protoplast were selected by 21% sucrose gradient, washed (10 mM HEPES, 5 mM CaCl_2_, 150 mM NaCl, 0.5 M manitol, pH 7) and conserved in a hormone solution (4.3 g/l MS salts, 0.5 M manitol, 1 mg/l 1-napthaleneacetic acid, 0.1 mg/l 6-benzylaminopurine, pH 5.8).

Analysis of the protoplasts was carried with a Gallios flow cytometer (Beckman Coulter). This instrument is equipped with a 488 nm/22 mW blue and a 405 nm/40 mW violet solid state diode lasers, two detectors for light scattering (forward scatter, *FS*, and side scatter, *SS*) and ten fluorescence detectors. *FS* measures cell size and *SS* defines protoplasts granularity. The *FS* signal was 10-fold reduced using a neutral density filter. The FL4 channel with a 670 nm band-pass was used to measure chlorophyll fluorescence. Live, intact protoplasts were selected based on the combination of *FS*, *SS* and FL4 reads. Venus and BFP contents on intact individual protoplasts were measured using the 525 nm (FL1) and the 450 nm (FL9) channels, respectively, in order to quantify the number of TEV-Venus, TEV-BFP and mix infected cells. For an example of the raw cytometry data see Figure S3.

### Data analysis and modeling of infection

#### Analysis of flow cytometry data

The combination of TagBFP [35] and Venus [34] was chosen in order to limit the overlap between excitation and emission spectra, allowing for the discrimination of the two fluorescent proteins. For *N. tabacum* plants infected with only TEV-Venus or TEV-BFP, we found low levels of background signal for the virus not present. The frequency of false-positive signals was low (mean frequency ± 1 SD): (6.29±3.08)×10^−4^ of cells gave Venus signal when the plant was only infected with TEV-BFP, whereas (7.74±5.26)×10^−3^ of cells gave a BFP signal when the plant was infected with only TEV-Venus. Since we were dealing with low levels of infection and to make our analysis conservative as possible, we decided to include an extra filter for background signal in the analysis of flow-cytometry data. From the data of non-infected or single-virus infected controls, we determined the 95 percentile, and used this point as the threshold for the extra filters. The *.lmd files were exported to *.csv format (GenePatternServer, http://www.broadinstitute.org/cancer/software/genepattern/), and all filtering of the data was then performed in the R 2.14 software with a custom script. The threshold values for filtering data were set to exclude dead cells, aggregated cells and limit false positive signal: *SS*≥56, *FS* Time of flight (TOF)≤310, Log_10_[FL4]≥45 (chlorophyll), Log_10_[FL1]>7.47 (TEV-Venus) and Log_10_[FL9]>37.39 (TEV-BFP).

The temporal increase in frequency of total infected cells (Figures 1A–D and 2A) for the pooled data was similar, but not identical, to measurements of virus genome copy numbers (Figure S1) [7], which validates that the frequency of infected cells as measured by flow cytometry is similar to a more classical measure of infection progress.

#### Estimates of cellular contagion rate (*R*)

To estimate the cellular contagion rate *R* we used an approach similar to Metcalf et al. [6], although in our case we are estimating the contagion rate per cell per day, rather than effective reproduction over the lifetime of a cell. We assume a Susceptible-Infectious (SI) model of within host dynamics at the cellular level. Recall that we proposed an SI meta-population model, in which *I_k_* is the fraction of infected cells and its change with time is given by Equation 1, *β* is the within-leaf transmission coefficient, *χ* is the between-leaf transmission coefficient, *S* is the fraction of susceptible cells and *k* denotes the leaf. For estimates of *R*, we considered only individual leaves, or the pooled data for the whole plant and disregarding any sub-partition into different leaves. We estimated the time-varying transmission constant *β_k,τ_* by predicting the infection level for the next data time point at which data were available (*τ*) using the recursive equation:
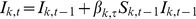
(2)over a period of *t*−*τ* days. *β_k,τ_* was estimated using a maximum likelihood approach to compare model predictions and the data, such that:
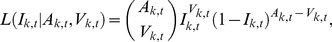
(3)where *A* is the total number of valid observations made by flow cytometry (the number of cells which pass all quality control filters), and *V* is the number of valid observations for which the cell was found to be infected by one or both virus variants. We performed a grid search over a large parameter space to obtain estimates of *β_k,τ_*, and also performed searches on 1000 bootstraps of the data to obtain confidence intervals for parameter estimates. 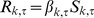 was then used to obtain *R* values for leaf *k*. Note that we did not incorporate aggregation of virus-infected cells in the estimate, in order to keep our estimates as close to the data as possible.

#### SI model fitting and selection

In this section five SI models are discussed, and we refer to the model described in the Results and Discussion section of the paper as Model 5.

Model 1 is the simplest variant of the model. The meta-population SI model is retained but the model does not allow for infection aggregation (*ψ* = 1), and therefore unlike Models 2–5, *S_k_* = 1−*I_k_*. The between-leaf transmission coefficient *χ* is assumed to be the same for Leaves 5–7. Note that no between-leaf transmission occurs for Leaf 3, because this is the inoculated leaf and phloem-based long-range viral movement is only to higher leaves. Three model parameters need to be estimated to fit the model: *I_1_*(0), the proportion of infected cells in the inoculated leaf at *t* = 0, *β* and *χ* (Table S1).

Model 2 introduces the infection aggregation parameter *ψ*, assuming it to be constant for all leaves. The model parameter *χ* is again assumed to be the same for Leaves 5–7, as in Model 1. Four model parameters need to be estimated to fit the model: *I_1_*(0), *β*, *χ* and *ψ* (Table S1).

Model 3 is an extension of Model 2, which allows *ψ* to be leaf dependent. Seven model parameters need to be estimated to fit the model: *I*_1_(0), *β*, *χ*, *ψ*_3_, *ψ*_5_, *ψ*_6_, and *ψ*_7_ (Table S1).

Model 4 is an extension of Model 2, which allows *χ* to be leaf dependent, while assuming *ψ* to be the same for each leaf. Note that because there is no within-leaves transmission to the inoculated leaf, *χ* estimates are only made for Leaves 2–4. Six model parameters need to be estimated to fit the model: *I*_1_(0), *β*, *χ*_5_, *χ*_6_, *χ*_7_ and *ψ* (Table S1).

Finally, Model 5 incorporates leaf-dependent infection aggregation and between-leaf transmission. Nine model parameters need to be estimated to fit the model: *I*_1_(0), *β*, *χ*_5_, *χ*_6_, *χ*_7_, *ψ*_3_, *ψ*_5_, *ψ*_6_, and *ψ*_7_ (Table S1).

Models were fitted using a maximum likelihood approach (Equation 3). We first performed grid searches over a large parameter space to ensure we had a global minimum for the negative log likelihood (NLL). Next, we performed stochastic hill climbing (SHC), initiating each hill climb from a random starting point in parameter space close to the global minimum for the NLL. Finally, to obtain 95% confidence intervals for model-parameter estimates, we repeated SHC on 1000 bootstraps of the data. Model parameter estimates and their 95% confidence intervals are given in Table S1. The Akaike Information Criterion (*AIC*) was used for model selection (Table S2).

#### *MOI* model fitting and selection

For model selection, we use the models and approach described in ref. [17], and briefly described below. These models are all based on the Poisson model, but incorporate a number of mechanisms that can account for deviations between the data and model predictions. The following notation is used: *m_I_* is the *MOI* in infected cells [7], with a range [1, ∞); *m_T_* is the *MOI* in all cells, including uninfected cells [9], and has a range [0, ∞); *p_A_* is the frequency of TEV-Venus (*A*), whereas *p_B_* is the frequency of TEV-BFP (*B*), which can be estimated as: 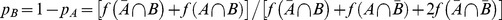, where *f*(·) represents the experimentally-observed frequencies of cells infected by none, one or both marked virus variants. We have not considered Model 1 [17], since it assumes an *MOI* that is constant over cells and gives similar results to Model 2, the null-model. Model 2 assumes the number of effectively infecting virions follows a Poisson distribution over all host cells. Model 3 incorporates spatial segregation of genotypes during virus expansion, by limiting the fraction of cells that can become coinfected. The rate of spatial segregation is determined by the parameter *φ* with range [0, ∞), and is non-existent if *φ* is zero and augments as *φ* becomes larger. Model 4 allows for the possibility that there is not perfect mixing, and that virus-infected cells tend to be aggregated. The model is therefore similar to SI Model 2, and again incorporates the infection aggregation parameter *ψ*:
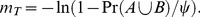
(4)where Pr(·) is the expected probability that cells are infected by none, one or both marked virus variants (as opposed to the observed frequency *f*(·)). Model 4a is a variant of Model 4 that assumes that each leaf has its own aggregation constant *ψ*. Four parameters must be estimated to fit the model: *ψ*_3_, *ψ*_5_, *ψ*_6_, and *ψ*_7_. Model 5 relaxes the assumption of independent action by virions and allows for superinfection exclusion [19], [38], [39], by relaxing assumptions about the relationship between the fraction of uninfected cells and *m_T_*. Parameter *ω* determines exclusion effects at *t* = 0, whilst *μ* controls the rate of change over time. As we expect only antagonistic effects (a lowering of the mean number of infecting virions per cell), the range of *ω* set to [0, 1] and *μ* to [0, ∞) [17]. We also combined the different models, in order to ascertain whether a combination of mechanisms could explain the data best (Table S3). Model 6 combines Models 2 and 4a; Model 7 combines Models 4a and 5; Model 8 combines Models 2 and 5; and Model 9 combines Models 2, 4a and 5. Model 4a was used instead of Model 4 in combination with other models because it incorporates the same mechanism and is better supported by the data.

In order to perform model fitting and selection, we exploit the fact that for each model there is a relationship between the fractions of uninfected and coinfected cells [17]. Model fitting (Table S3) was performed as above. The likelihood of a particular probability of coinfection is then:
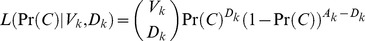
(5)where *D_k_* is the number of coinfected cells observed (and *V_k_* is again the number of infected cells). Model selection was again performed using *AIC* (Table S4). For the best fitting model (Model 4a), we predicted *MOI* (*m_T_*) with Equation 4. To calculate *m_I_* values from *m_T_*, we used the relationship between the means of a zero-truncated and a non-truncated Poisson distribution [17], [40]: 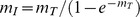.

#### Analysis of variation in genotype frequencies

In order to estimate effective population sizes, we used *F_ST_* statistics [41] as described in Monsion et al. [25]. To estimate *N_e_* in the whole plant and in the inoculated leaf (Leaf 3), we assumed *H_T_* – the genetic diversity under the assumption all subpopulation form a single population – was zero, given that the inoculum dose administered is very high and as such we do not expect there will any appreciable differences in the frequencies of the genotypes over inoculated leaves (and therefore over plants). For calculating *F_ST_* values for Leaves 5, 6 and 7, we calculated *H_T_* for the inoculated leaf, given that this leaf is the sole source for infection in Leaf 5 and the chief source for infection in Leaves 6 and 7. The SI model (Figure 2) indicates there is virtually no infection in Leaf 5, meaning it will probably not appreciably contribute to infection in Leaf 6. Leaf 6 may contribute some to infection in Leaf 7, but in this case using only Leaf 3 to calculate *H_T_* is a reasonable approximation nonetheless, since there is no significant bottleneck between Leaves 3 and 7. We estimate *N_e_* for each leaf at each time point, and for the pooled data of days 5, 7 and 10 (Figure S2), in order to increase the power of our estimates and because the genetic diversity should not change once infection has been established. Day 3 was excluded because infection levels in some leaves are very low and thereby increase diversity to levels that are probably not representative of that caused by the genetic bottleneck into the leaf alone.

## Supporting Information

Figure S1 Comparison of infected cells to genome copy numbers is shown. Frequency of infected cells (ordinate) over time (abscissae) is given, for the data pooled from all leaves (black circles). Each data point represents the mean of 5 plants and error bars indicate ± 1 SD. The black line shows a logistic model fitted to the data, whereas the brown line indicates a logistic growth curve fitted to RT-qPCR data in a similar experiment [7], with the data scaled so that *κ* values (the carry capacity) are the same. The RT-qPCR-based curve is surprisingly similar to infected-cell curve, although at 5 dpi predicted TEV RNA levels appear to be relatively lower than the proportion of infected cells. This discrepancy may depend on the methodology used, or there may be a large number of cells that are in early infection, when the fluorescent marker protein is expressed but viral RNA accumulation levels are still low.
(TIF)

Figure S2 *N_e_* estimates for different leaves and times. Whereas Figure 5B represents estimates of *N_e_*, the effective population size, for pooled data of days 5, 7 and 10, here we provide estimates for the data from individual days, with error bars indicating the 95% CI. In Panels A–D the data for Leaves 3, 5, 6 and 7 are given, respectively, and Panel E provides the pooled data of all leaves. Bars or error bars that extend to the top of the panel indicate values extending to ∞, whereas for Leaf 3 day 3 the lower limit of the CI is 1. Each data point is the mean of 5 plants.
(TIF)

Figure S3 Flow cytometry data. Histograms showing the fluorescence measurement events on four different channels, with example data from one replicate of Leaf 6 at 7 dpi. In all four panels, the red line is the threshold value used in the data analysis. For panels A and B, the data for all 50,000 counts made are given. In panel A, the log-transformed fluorescence measured on the side scatter channel is given. Side scatter depends on the granularity of the cells, and is therefore an indication of the viability of a cell. In panel B, the log-transformed fluorescence on the FL4 channel is given, which correlates to the chlorophyll content of the cell and therefore indicates intact cells. For both side scatter and chlorophyll content, there is a clear separation between the selected and excluded measurements. In Panels C and D, we give only measurements that passed through initial filtering, meeting criteria for side scatter, chlorophyll and time of flight. In Panel C, the log-transformed fluorescence on the FL1 channel is given, which corresponds to the Venus marker protein, whereas in panel D we give the log-transformed fluorescence on FL9, corresponding to BFP. Even in a leaf with relatively high infection levels, the majority of cells give the minimum fluorescence level (set by default to log_10_[0.1024] = −0.9897). The cutoff values are clearly conservative with respect to determining whether cells are infected (see Materials and Methods for details).
(TIF)

Table S1 Estimated model parameters for SI models.
(DOCX)

Table S2 Model selection for SI models.
(DOCX)

Table S3 Estimated *MOI* model parameters.
(DOCX)

Table S4 *MOI* model selection.
(DOCX)

## References

[1] Grenfell BT, Pybus OG, Gog JR, Wood JLN, Daly JM, et al (2004) Unifying the epidemiological and evolutionary dynamics of pathogens. Science 303: 327–332. 14726583 10.1126/science.1090727

[2] Mideo N, Alizon S, Day T (2008) Linking within- and between-host dynamics in the evolutionary epidemiology of infectious diseases. Trends Ecol Evol 23: 511–517. 18657880 10.1016/j.tree.2008.05.009

[3] Martínez F, Sardanyés J, Elena SF, Daròs JA (2011) Dynamics of a plant RNA virus intracellular accumulation: Stamping machine vs. geometric replication. Genetics 188: 637–646. 21515574 10.1534/genetics.111.129114 PMC3176528

[4] Cuevas JM, Moya A, Sanjuán R (2005) Following the very initial growth of biological RNA viral clones. J Gen Virol 86: 435–443. 15659763 10.1099/vir.0.80359-0

[5] Dolja VV, McBride HJ, Carrington JC (1992) Tagging of plant potyvirus replication and movement by insertion of β-glucuronidase into the viral polyprotein. Proc Natl Acad Sci USA 89: 10208–10212. 1438210 10.1073/pnas.89.21.10208 PMC50307

[6] Metcalf CJE, Graham AL, Huijben S, Barclay VC, Long GH, et al (2011) Partitioning regulatory mechanisms of within-host malaria dynamics using the effective propagation number. Science 333: 984–988. 21852493 10.1126/science.1204588 PMC3891600

[7] González-Jara P, Fraile A, Canto T, García-Arenal F (2009) The multiplicity of infection of a plant virus varies during colonization of its eukaryotic host. J Virol 83: 7487–7494. 19474097 10.1128/JVI.00636-09 PMC2708614

[8] Zwart MP, Daròs JA, Elena SF (2012) Effects of potyvirus effective population size in inoculated leaves on viral accumulation and the onset of symptoms. J Virol 86: 9737–9747. 22740417 10.1128/JVI.00909-12 PMC3446627

[9] Gutiérrez S, Yvon M, Pirolles E, Garzo E, Fereres A, et al (2010) Dynamics of the multiplicity of cellular infection in a plant virus. PLoS Pathog 6: e1001113. 20862320 10.1371/journal.ppat.1001113 PMC2940754

[10] Li HY, Roossinck MJ (2004) Genetic bottlenecks reduce population variation in an experimental RNA virus population. J Virol 78: 10582–10587. 15367625 10.1128/JVI.78.19.10582-10587.2004 PMC516416

[11] Sacristán S, Malpica JM, Fraile A, García-Arenal F (2003) Estimation of population bottlenecks during systemic movement of *Tobacco mosaic virus* in tobacco plants. J Virol 77: 9906–9911. 12941900 10.1128/JVI.77.18.9906-9911.2003 PMC224588

[12] Bald JG (1937) The use of numbers of infections for comparing the concentration of plant virus suspensions I. Dilution experiments with purified suspensions. Ann Appl Biol 24: 33–55.

[13] Miyashita S, Kishino H (2010) Estimation of the size of genetic bottlenecks in cell-to-cell movement of *Soil-borne wheat mosaic* virus and the possible role of the bottlenecks in speeding up selection of variations in trans-acting genes or elements. J Virol 84: 1828–1837. 19955302 10.1128/JVI.01890-09 PMC2812364

[14] Zwart MP, Daròs JA, Elena SF (2011) One is enough: *In vivo* effective population size is dose-dependent for a plant RNA virus. PLoS Pathog 7: e1002122. 21750676 10.1371/journal.ppat.1002122 PMC3131263

[15] Lafforgue G, Tromas N, Elena SF, Zwart MP (2012) Dynamics of the establishment of systemic *Potyvirus* infection: Independent yet cumulative action of primary infection sites. J Virol 86: 12912–12922. 22993154 10.1128/JVI.02207-12 PMC3497618

[16] González-Jara P, Fraile A, Canto T, García-Arenal F (2013) Author's correction: The multiplicity of infection of a plant virus varies during colonization of its eukaryotic host. J Virol 87: 2374. 10.1128/JVI.00636-09 PMC2708614 19474097

[17] Zwart MP, Tromas N, Elena SF (2013) Model-selection-based approach for calculating cellular multiplicity of infection during virus colonization of multi-cellular hosts. PLoS ONE 8: e64657. 23724074 10.1371/journal.pone.0064657 PMC3665715

[18] Gutiérrez S, Yvon M, Pirolles E, Garzo E, Fereres A, et al (2012) Circulating virus load determines the size of bottlenecks in viral populations progressing within a host. PLoS Pathog 8: e1003009. 23133389 10.1371/journal.ppat.1003009 PMC3486874

[19] Dietrich C, Maiss E (2003) Fluorescent labelling reveals spatial separation of *Potyvirus* populations in mixed infected *Nicotiana benthamiana* plants. J Gen Virol 84: 2871–2876. 13679622 10.1099/vir.0.19245-0

[20] Sankara Rao K, Prakash AH (1995) A simple method for the isolation of plant protoplasts. J Biosci 20: 645–655.

[21] Zwart MP, Willemsen A, Daròs JA, Elena SF (in press) Experimental evolution of pseudogenization and gene loss in a plant RNA virus. Mol Biol Evol 31 1: 121–134. 24109604 10.1093/molbev/mst175 PMC3879446

[22] Roberts AG, Cruz SS, Roberts IM, Prior DAM, Turgeon R, et al (1997) Phloem unloading in sink leaves of *Nicotiana benthamiana*: Comparison of a fluorescent solute with a fluorescent virus. Plant Cell 9: 1381–1396. 12237387 10.1105/tpc.9.8.1381 PMC157005

[23] Barlow ND (1991) A spatially aggregated disease host model for bovine TB in New Zealand possum populations. J Appl Ecol 28: 777–793.

[24] Barlow ND (2000) Non-linear transmission and simple models for bovine tuberculosis. J Animal Ecol 69: 703–713.

[25] Monsion B, Froissart R, Michalakis Y, Blanc S (2008) Large bottleneck size in *Cauliflower mosaic virus* populations during host plant colonization. PLoS Pathog 4: e1000174. 18846207 10.1371/journal.ppat.1000174 PMC2553192

[26] Hall JS, French R, Hein GL, Morris TJ, Stenger DC (2001) Three distinct mechanisms facilitate genetic isolation of sympatric *Wheat streak mosaic virus* lineages. Virology 282: 230–236. 11289805 10.1006/viro.2001.0841

[27] Waterhouse PM, Wang MB, Lough T (2001) Gene silencing as an adaptive defence against viruses. Nature 411: 834–842. 11459066 10.1038/35081168

[28] Voinnet O, Vain P, Angell S, Baulcombe DC (1998) Systemic spread of sequence-specific transgene RNA degradation in plants is initiated by localized introduction of ectopic promoterless DNA. Cell 95: 177–187. 9790525 10.1016/s0092-8674(00)81749-3

[29] Paschalidis AP, Roubelakis-Angelakis KA (2005) Spatial and temporal distribution of polyamine levels and polyamine anabolism in different organs/tissues of the tobacco plant. Correlations with age, cell division/expansion, and differentiation. Plant Physiol 138: 142–152. 15849310 10.1104/pp.104.055483 PMC1104170

[30] Keeling MJ (1999) The effects of local spatial structure on epidemiological invasions. Proc R Soc B 266: 859–867. 10.1098/rspb.1999.0716 PMC1689913 10343409

[31] van Baalen M, Sabelis MW (1995) The milker-killer dilemma in spatially structured predator-prey interactions. Oikos 74: 391–400.

[32] Boots M, Mealor M (2007) Local interactions select for lower pathogen infectivity. Science 315: 1284–1286. 17332415 10.1126/science.1137126

[33] Bedoya LC, Daròs JA (2010) Stability of *Tobacco etch virus* infectious clones in plasmid vectors. Virus Res 149: 234–240. 20152868 10.1016/j.virusres.2010.02.004

[34] Nagai T, Ibata K, Park ES, Kubota M, Mikoshiba K, et al (2002) A variant of yellow fluorescent protein with fast and efficient maturation for cell-biological applications. Nat Biotechnol 20: 87–90. 11753368 10.1038/nbt0102-87

[35] Subach OM, Gundorov IS, Yoshimura M, Subach FV, Zhang J, et al (2008) Conversion of red fluorescent protein into a bright blue probe. Chem Biol 15: 1116–1124. 18940671 10.1016/j.chembiol.2008.08.006 PMC2585067

[36] Carrasco P, Daròs JA, Agudelo-Romero P, Elena SF (2007) A real-time RT-PCR assay for quantifying the fitness of *Tobacco etch virus* in competition experiments. J Virol Meth 139: 181–188. 10.1016/j.jviromet.2006.09.020 17092574

[37] Majer E, Daròs JA, Zwart MP (2013) Stability and fitness impact of the visually discernable Rosea1 marker in the *Tobacco etch virus* genome. Viruses 5: 2153–2168. 24022073 10.3390/v5092153 PMC3798895

[38] Kunkel LO (1934) Studies on acquired immunity with tobacco and aucuba mosaic. Phytopathology 24: 437–66.

[39] Thung TH (1928) Physiologisch onderzoek met betrekking tot het virus der bladrolziekte van de aardappel-plant, *Solanum tuberosum* L. Tijdschrift over Plantenziekten 34: 1–74.

[40] Olkin I, Gleser, L.J. Derman, C. (1994) Probability Models and Applications, 2^nd^ ed. New York: Macmillan. 575 p.

[41] Wright S (1931) Evolution in Mendelian populations. Genetics 16: 97–159. 17246615 10.1093/genetics/16.2.97 PMC1201091

