# Supplementary figures and images for: Within-Host Spatiotemporal Dynamics of Plant Virus Infection at the Cellular Level
Source: PLoS Genet. 2014 Feb 27;10(2):e1004186. doi: 10.1371/journal.pgen.1004186 (PMC3937225; doi:10.1371/journal.pgen.1004186)

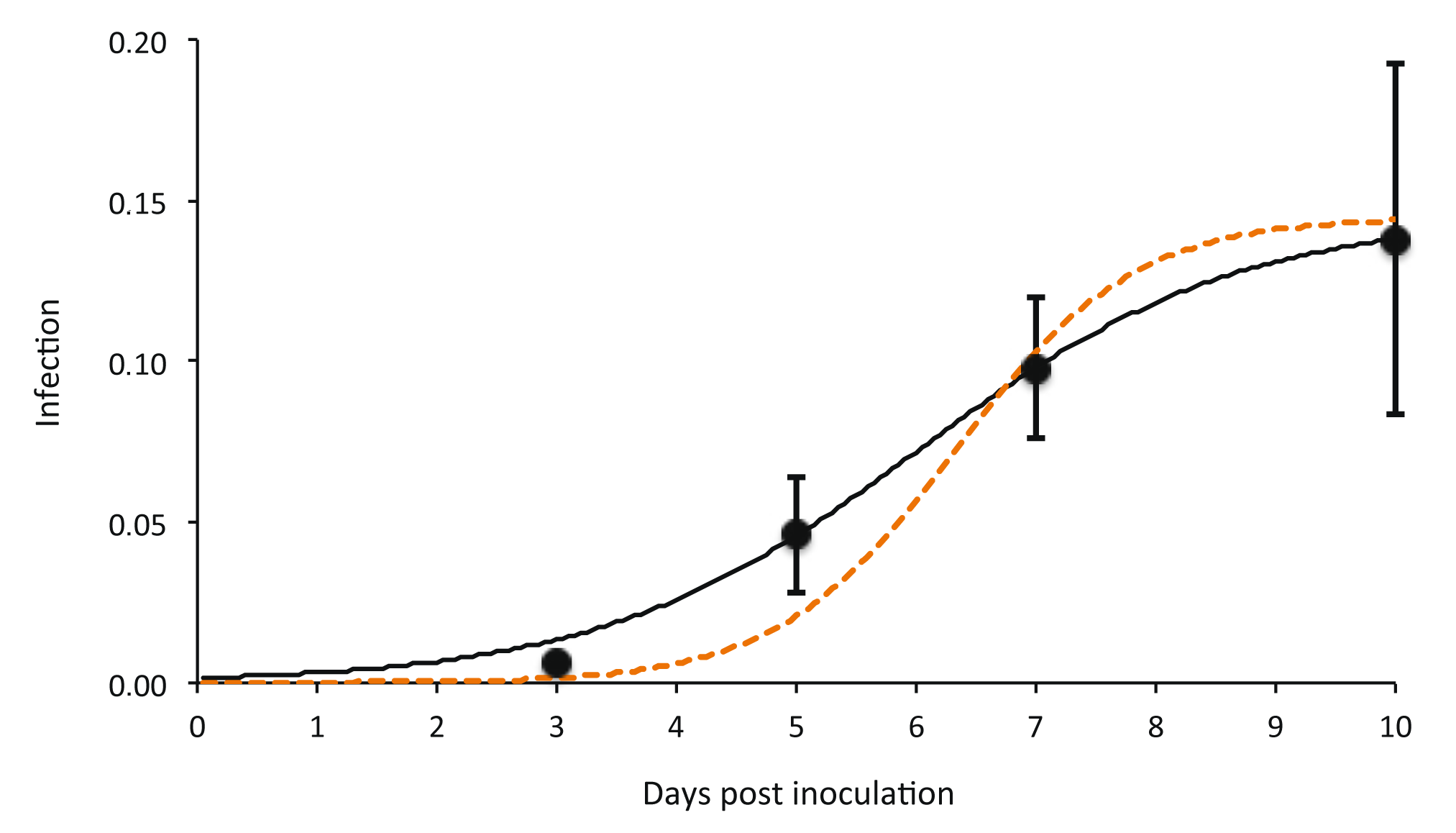

Supplement: Figure S1 — Comparison of infected cells to genome copy numbers is shown. Frequency of infected cells (ordinate) over time (abscissae) is given, for the data pooled from all leaves (black circles). Each data point represents the mean of 5 plants and error bars indicate ± 1 SD. The black line shows a logistic model fitted to the data, whereas the brown line indicates a logistic growth curve fitted to RT-qPCR data in a similar experiment [7], with the data scaled so that κ values (the carry capacity) are the same. The RT-qPCR-based curve is surprisingly similar to infected-cell curve, although at 5 dpi predicted TEV RNA levels appear to be relatively lower than the proportion of infected cells. This discrepancy may depend on the methodology used, or there may be a large number of cells that are in early infection, when the fluorescent marker protein is expressed but viral RNA accumulation levels are still low. (TIF) [file pgen.1004186.s001.tif]

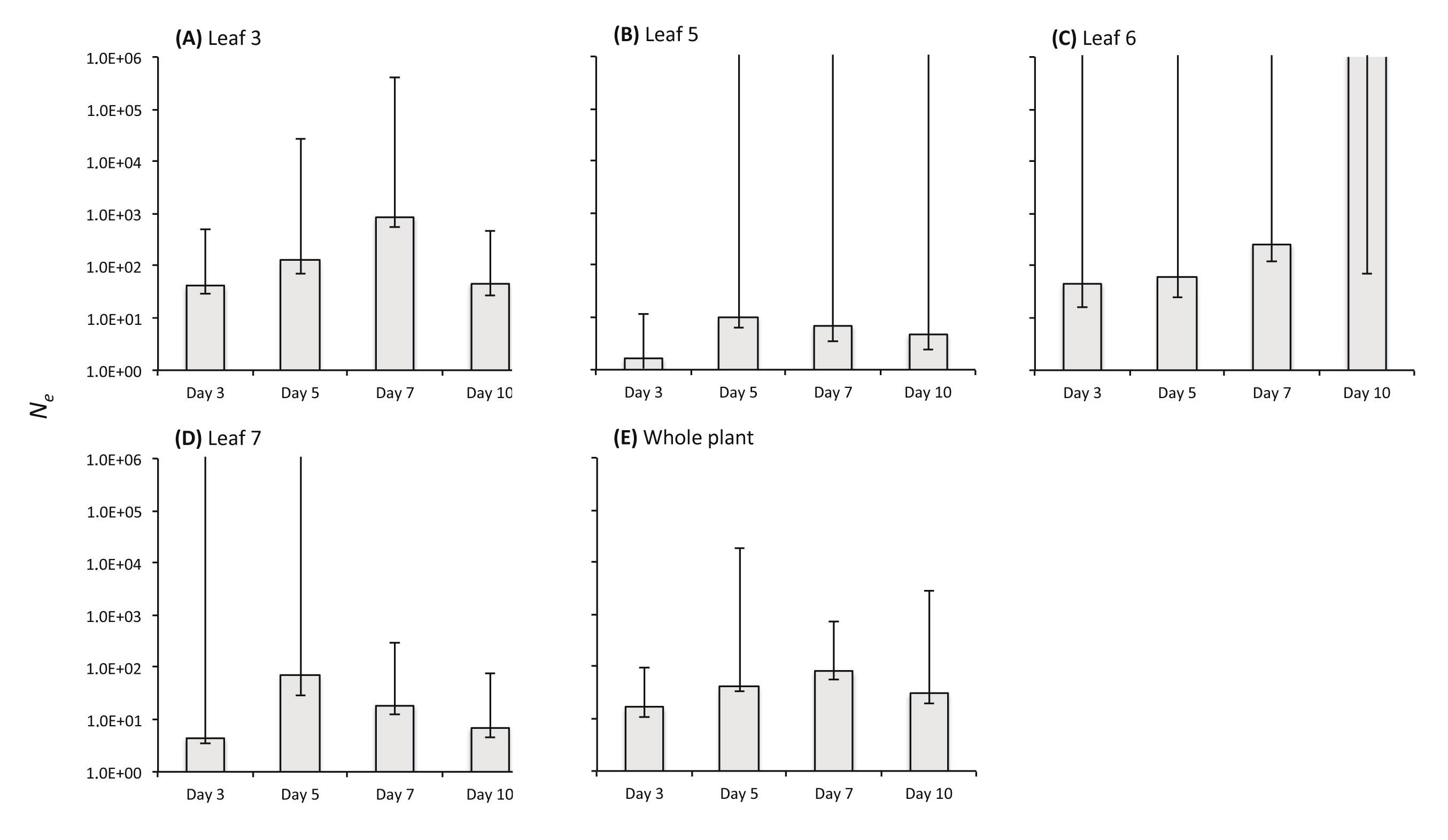

Supplement: Figure S2 — Ne estimates for different leaves and times. Whereas Figure 5B represents estimates of Ne, the effective population size, for pooled data of days 5, 7 and 10, here we provide estimates for the data from individual days, with error bars indicating the 95% CI. In Panels A–D the data for Leaves 3, 5, 6 and 7 are given, respectively, and Panel E provides the pooled data of all leaves. Bars or error bars that extend to the top of the panel indicate values extending to ∞, whereas for Leaf 3 day 3 the lower limit of the CI is 1. Each data point is the mean of 5 plants. (TIF) [file pgen.1004186.s002.tif]

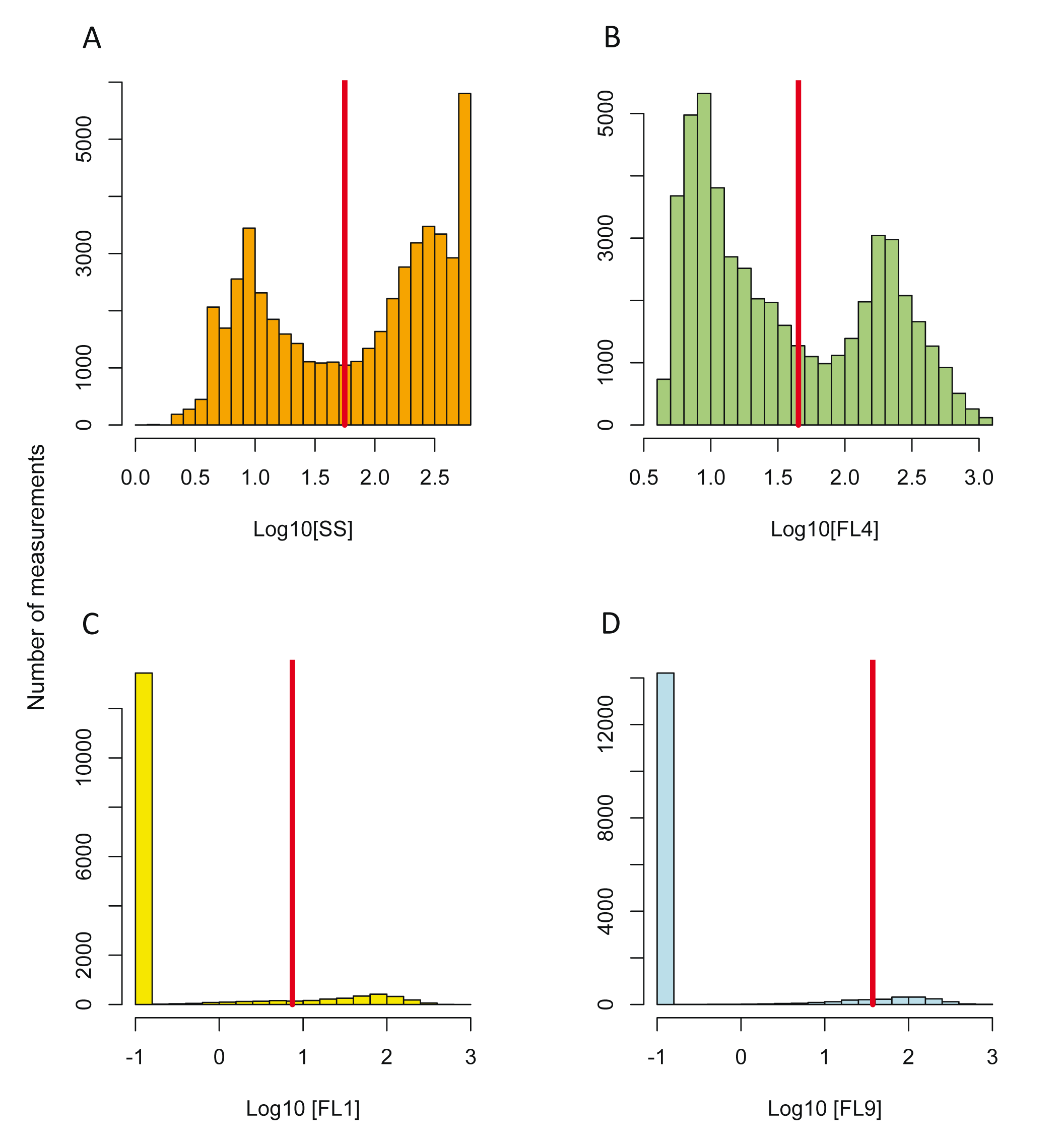

Supplement: Figure S3 — Flow cytometry data. Histograms showing the fluorescence measurement events on four different channels, with example data from one replicate of Leaf 6 at 7 dpi. In all four panels, the red line is the threshold value used in the data analysis. For panels A and B, the data for all 50,000 counts made are given. In panel A, the log-transformed fluorescence measured on the side scatter channel is given. Side scatter depends on the granularity of the cells, and is therefore an indication of the viability of a cell. In panel B, the log-transformed fluorescence on the FL4 channel is given, which correlates to the chlorophyll content of the cell and therefore indicates intact cells. For both side scatter and chlorophyll content, there is a clear separation between the selected and excluded measurements. In Panels C and D, we give only measurements that passed through initial filtering, meeting criteria for side scatter, chlorophyll and time of flight. In Panel C, the log-transformed fluorescence on the FL1 channel is given, which corresponds to the Venus marker protein, whereas in panel D we give the log-transformed fluorescence on FL9, corresponding to BFP. Even in a leaf with relatively high infection levels, the majority of cells give the minimum fluorescence level (set by default to log10[0.1024] = −0.9897). The cutoff values are clearly conservative with respect to determining whether cells are infected (see Materials and Methods for details). (TIF) [file pgen.1004186.s003.tif]
