# Supplementary material for: Within-Host Spatiotemporal Dynamics of Plant Virus Infection at the Cellular Level
Source: PLoS Genet. 2014 Feb 27;10(2):e1004186. doi: 10.1371/journal.pgen.1004186 (PMC3937225; doi:10.1371/journal.pgen.1004186)
Supplement: Table S1 — Estimated model parameters for SI models. (DOCX) [file pgen.1004186.s004.docx]

**Table S1.** Estimated model parameters for SI models.

| Model | Parameter estimates [95% CI] |
| --- | --- |
| 1 | *I*_1_(0) = 1.43 [1.27-1.60] × 10^−2^  *β* = 3.46 [1.58-5.89] × 10^−3^  *χ* = 6.76 [5.62-7.94] × 10^−3^ |
| 2 | *I*_1_(0) = 1.86 [0.63-3.89] × 10^−2^  *β* = 7.24 [2.95-8.51] × 10^−3^  *χ* = 9.12 [7.08-13.18] × 10^−3^  *ψ* = 0.345 [0.206-0.445] |
| 3 | *I*_1_(0) = 1.82 [1.18-56.23] × 10^−3^  *β =* 4.07 [1.05-5.01] × 10^−2^  *χ* = 1.82 [1.17-11.48] × 10^−3^  *ψ*_3_ = 0.069 [0.053-0.196]  *ψ*_5_ = 0.006 [0.006-0.096]  *ψ*_6_ = 0.265 [0.158-0.393]  *ψ*_7_ = 0.225 [0.157-0.394] |
| 4 | *I*_1_(0) = 2.82 [1.23-10.24] × 10^−3^  *β =* 1.73 [1.07-2.69] × 10^−2^  *χ_5_* = 1.12 [0.31-2.09] × 10^−3^  *χ_6_ =* 5.89 [2.82-8.51] × 10^−2^  *χ_7_ =* 4.27 [2.13-8.91] × 10^−3^  *ψ* = 0.274 [0.155-0.345] |
| 5 | *I*_1_(0) = 3.72 [1.74-17.38] × 10^−3^  *β =* 0.871 [0.257-1.660]  *χ_5_* = 0.724 [0.033-0.813]  *χ_6_ =* 1.38 [0.58-2.34]  *χ_7_ = 0.*107 [0.050-0.263]  *ψ*_3_ = 0.083 [0.053-0.147]  *ψ*_5_ = 0.018 [0.002-0.050]  *ψ*_6_ = 0.233 [0.155-0.345]  *ψ*_7_ = 0.286 [0.234-0.346] |
