# Supplementary material for: Within-Host Spatiotemporal Dynamics of Plant Virus Infection at the Cellular Level
Source: PLoS Genet. 2014 Feb 27;10(2):e1004186. doi: 10.1371/journal.pgen.1004186 (PMC3937225; doi:10.1371/journal.pgen.1004186)
Supplement: Table S2 — Model selection for SI models. (DOCX) [file pgen.1004186.s005.docx]

**Table S2.** Model selection for SI models.

| Model | Parameters | NLL | *AIC* | *ΔAIC* | *AW* |
| --- | --- | --- | --- | --- | --- |
| 1 | 3 | 49579.85 | 99165.70 | 57329,80 | 0 |
| 2 | 4 | 48158.28 | 96234,55 | 54488.65 | 0 |
| 3 | 7 | 36827.68 | 73669.36 | 31833.46 | 0 |
| 4 | 6 | 25695.90 | 51403.79 | 9567.89 | 0 |
| 5 | 9 | 21423.69 | 41835,90 | - | 1 |

Results of model selection using the Akaike Information Criterion (*AIC*). Model refers to the SI Model number in Materials and Methods, with Model 5 being the model discussed in the results section of the paper. Parameters is the number of model parameters, NLL is the negative log likelihood, *ΔAIC* is the difference in *AIC* between the model in question and the best fitting model (Model 5), and *AW* is the Akaike Weight, which gives an indication of the probability that a model is best supported.
