# Supplementary material for: Within-Host Spatiotemporal Dynamics of Plant Virus Infection at the Cellular Level
Source: PLoS Genet. 2014 Feb 27;10(2):e1004186. doi: 10.1371/journal.pgen.1004186 (PMC3937225; doi:10.1371/journal.pgen.1004186)
Supplement: Table S3 — Estimated MOI model parameters. (DOCX) [file pgen.1004186.s006.docx]

**Table S3.** Estimated *MOI* model parameters.

| Model | Parameter estimates [95% CI] |
| --- | --- |
| 2 | - |
| 3 | *ϕ* = 0 [0-0.029] |
| 4 | *ψ* = 0.522 [0.433-0.627] |
| 4a | *ψ*_3_ = 0.222 [0.191-0.262]  *ψ*_5_ = 0.125 [0.071-0.138]  *ψ*_6_ = 0.508 [0.368-0.536]  *ψ*_7_ = 0.544 [0.448-0.597] |
| 5 | *ω* = 1 [*]  *μ* = 0 [*] |
| 6 | *ϕ* = 0 [0-0.026]  *ψ*_3_ = 0.222 [0.182-0.259]  *ψ*_5_ = 0.125 [0.071-0.135]  *ψ*_6_ = 0.508 [0.368-0.531]  *ψ*_7_ = 0.544 [0.437-0.609] |
| 7 | *ω* = 1 [*]  *μ* = 0 [0-0.018]  *ψ*_3_ = 0.222 [0.187-0.260]  *ψ*_5_ = 0.125 [0.071-0.133]  *ψ*_6_ = 0.508 [0.368-0.535]  *ψ*_7_ = 0.544 [0.438-0.595] |
| 8 | *ϕ* = 0 [0-0.005]  *ω* = 1 [*]  *μ* = 0 [0-0.017] |
| 9 | *ϕ* = 0 [0-0.026]  *ω* = 1 [*]  *μ* = 0 [0.001]  *ψ*_3_ = 0.222 [0.183-0.260]  *ψ*_5_ = 0.125 [0.071-0.135]  *ψ*_6_ = 0.508 [0.368-0.532]  *ψ*_7_ = 0.544 [0.436-0.611] |

***** indicates the lower and upper 95% CI limits coincide with the estimate parameter value.
