# Supplementary material for: Within-Host Spatiotemporal Dynamics of Plant Virus Infection at the Cellular Level
Source: PLoS Genet. 2014 Feb 27;10(2):e1004186. doi: 10.1371/journal.pgen.1004186 (PMC3937225; doi:10.1371/journal.pgen.1004186)
Supplement: Table S4 — MOI model selection. (DOCX) [file pgen.1004186.s007.docx]

**Table S4.** *MOI* model selection.

| Model | Parameters | NLL | *AIC* | *ΔAIC* | *AW* |
| --- | --- | --- | --- | --- | --- |
| 2 | 0 | 14261.17 | 28522.34 | 22569.22 | 0 |
| 3 | 1 | 14261.17 | 28524.34 | 22571.22 | 0 |
| 4 | 1 | 3567.58 | 7137.15 | 1184.03 | 0 |
| 4a | 4 | 2972.56 | 5953.12 | - | 0.644 |
| 5 | 2 | 14261.17 | 28526.34 | 22573.22 | 0 |
| 6 | 5 | 2972.56 | 5955.12 | 2.00 | 0.237 |
| 7 | 6 | 2972.56 | 5957.12 | 4.00 | 0.087 |
| 8 | 3 | 14261.17 | 28528.34 | 22575.22 | 0 |
| 9 | 7 | 2972.56 | 5959.124 | 6.00 | 0.032 |

Results of model selection using the Akaike Information Criterion (*AIC*). Model refers to the model number in Materials and Methods and [23], with Model 4a being the best-supported model and the model discussed in the results section of the paper. Parameters is the number of model parameters, NLL is the negative log likelihood, *ΔAIC* is the difference in *AIC* between the model in question and the best fitting model, and *AW* is the Akaike Weight, which gives an indication of the probability that a model is best supported. Although Models 6, 7 and 9 have similar *AIC* values to Model 4a and an appreciable *AW*, these models can be discounted since at the estimated parameter values (Table S3) these models collapse to Model 4a.
